# Lunch Salad Bars in New Orleans’ Middle and High Schools: Student Intake of Fruit and Vegetables

**DOI:** 10.3390/ijerph14040415

**Published:** 2017-04-13

**Authors:** Carolyn C. Johnson, Leann Myers, Adrienne R. Mundorf, Keelia O’Malley, Lori Andersen Spruance, Diane M. Harris

**Affiliations:** 1 Department of Global Community Health and Behavioral Sciences, School of Public Health and Tropical Medicine, Tulane University, New Orleans, LA 70112, USA; komalley@tulane.edu; 2 Department of Global Biostatistics and Informatics, School of Public Health and Tropical Medicine, Tulane University, New Orleans, LA 70112, USA; myersl@tulane.edu; 3 Sisters of Charity Foundation of Cleveland, Cleveland, OH 44115, USA; amundorf@socfcleveland.org; 4 Department of Health Sciences, Brigham Young University, Provo, UT 84602, USA; lori.andersen@byu.edu; 5 Division of Nutrition, Physical Activity and Obesity, Centers for Disease Control and Prevention, Atlanta, GA 30329, USA; dmharris@cdc.gov

**Keywords:** lunch salad bars, school environment, fruit and vegetable consumption, school lunch, adolescent, diet, school nutrition service

## Abstract

The school lunch salad bar (SB) is a recommended food environmental strategy to increase access to, and consumption of fruit and vegetables (F/V). In a study to examine use of school lunch SBs, middle and high school students provided data via the Automated Self-Administered 24-h dietary recall (24HDR) tool for kids (ASA24-Kids-2012), a web-based data collection platform. Kilocalories were computed, food groups were assigned and F/V sources were obtained. Students (n = 718) from 12 schools with SBs and nine schools without SBs were approximately 87% African American, over 64% female and most were 7th and 8th graders. SB school students had higher median energy consumption at lunch but a higher percent of non-SB students reported eating fruit at lunch compared to SB students. Most students reporting eating F/V at lunch obtained F/V from the cafeteria main line; only 19.6% reported eating F/V exclusively from the SB. In SB schools median intake of cups F/V was higher among students using the SB (0.92) compared to those not using the SB (0.53). Results of this study are mixed, but encouraging. Additional factors, e.g., nutrition education, marketing, and kinds of foods offered on the SB need to be examined for potential influence on SB use.

## 1. Introduction

The high prevalence of adolescent overweight and obesity in the United States is a serious public health problem. In 2011–2012 the prevalence of overweight and obesity combined exceeded one-third of the adolescent population [1]. The problem is especially prominent in African-American adolescents, with nearly 40% of this population being overweight or obese [1]. Adolescent overweight and obesity are linked to numerous health consequences, including cardiovascular disease, hypertension and insulin resistance/type 2 diabetes [2,3,4]. Furthermore, the likelihood of overweight or obesity persisting into adulthood is higher among overweight/obese adolescents [5,6,7].

Unhealthy dietary behaviors are a contributing factor to overweight/obesity, as well as to increased risk for related chronic diseases among adolescents. Over-consumption of energy-dense snack foods and beverages has been linked to adolescent overweight and obesity [8] and increased risk for high blood pressure and type 2 diabetes [9]. To promote less energy-dense foods in adolescent diets, the 2015 Dietary Guidelines recommended that daily consumption include more fruit and vegetables (F/V) [10]. National surveys, however, have found that adolescent consumption of fruit, and particularly vegetables, is much lower than the recommendations in the 2010 Dietary Guidelines for Americans [11], which recommendations are the same as the 2015 Dietary Guidelines. Because adolescents spend most of their time in school and over one-third of their daily energy intake occurs during school hours, the school food environment is especially influential on children’s and adolescents’ diets, and efforts have been made to increase access to F/V within the school food environment [12,13].

Introducing school salad bars (SBs) stocked with F/V is a recommended strategy to increase access to and promote increased consumption of F/V among adolescents [14,15,16]. Studies have shown that school SBs have actually increased access to, as well as variety of, F/V offered to students as part of the school lunch program [17]. The Let’s Move Salad Bars to Schools (LMSBS) program promotes SBs by providing SB equipment to schools to supplement the food environment to increase access to F/V among schoolchildren [18].

Several studies have investigated the association between SBs and F/V consumption among students in elementary schools. A cross-sectional study conducted among elementary school students using 24-h dietary recalls (24HDR) found a marked increase in F/V consumption after the introduction of SBs and nutritional education and promotional components [19]. A cross-sectional plate waste study, also among elementary school students, found that, while F/V consumption increased significantly as the variety of F/V offerings increased, the presence of an SB was not associated with higher F/V consumption compared to the presence of pre-portioned F/V servings [20].

The relationship between school SB presence and F/V consumption is less studied among middle and high school students. A national study found that, as SB accessibility rose among middle school students, green vegetable consumption increased. This study, however, involved a student self-reported food frequency questionnaire to measure consumption and no comparison group was involved [21].

In order to obtain more information about the impact of SBs in middle and high schools, we conducted a study to determine if higher F/V consumption among students was associated with the presence of an SB in those school cafeterias. An additional objective of this study was to examine the contribution of F/V consumption at lunch relative to intake for the rest of the day.

## 2. Materials and Methods

### 2.1. Study Design

The data for these analyses were collected within a school-based study that used a multi-stakeholder cross-sectional design to examine schools with SBs and schools without SBs. The LMSBS campaign donated SB units to 43 New Orleans, Louisiana, public schools by request of the schools [22]. SB study eligibility included Orleans Parish schools that contained at least one grade each at the 7th through 12th grade levels, were recipients of donated SB units from the LMSBS initiative, and did not participate in a previous pilot study on SB use. Non-SB school eligibility was the same, except that these schools did NOT have SB units, but were similar to SB schools in average standardized test scores (85% SB schools; 83% non-SB schools); racial distribution (88% African American SB schools; 94% African American non-SB schools), and percentage of student population receiving free or reduced lunch (85% SB schools; 90% non-SB schools). Consequently, the data were collected from 12–20 years old adolescents from 12 SB schools and nine non-SB schools. The Tulane University Social and Behavioral Institutional Review Board approved the research for this project.

### 2.2. Participants

Detailed methods are described in Andersen et al. [22]. Twenty-one schools participated in this study, with a total enrollment for 7th and 8th graders of 1942 students and a total enrollment of 3005 for high school students. The eligible pool of students for the 24HDR consisted of 7th through 12th grade students who completed the self-report student survey with passive parental consent (n = 1069 students from SB schools; n = 909 students from non-SB schools). Recruitment for the dietary recalls occurred in the classrooms among eligible students and required active parental consents. All students who participated previously in the self-report survey, and provided active parental consent as well as student assents were eligible to participate in the 24HDR. Students were not randomized but were immediately enrolled in the 24HDR study when they produced the active parental consent. This continued until it was clear that no additional active parental consents would be forthcoming; therefore, all eligible students who delivered an active parental consent were enrolled in the 24HDR.

### 2.3. Data Collection

Tulane University graduate student research assistant (RAs) were trained to conduct a single 24-h dietary recall (24HDR) with each participating student at the school site. The Automated Self-Administered 24HDR tool for kids (ASA24-Kids-2012) is a web-based data collection platform developed by the National Cancer Institute. It uses the validated US Department of Agriculture (USDA) Automated Multiple-Pass Method to guide participants through seven stages of data collection [22,23,24,25,26]. The ASA24-Kids-2012 platform collects complete dietary intake information from the 24-h period of the previous day (midnight to midnight) and uses the USDA Food and Nutrient Database for Dietary Studies version 4.1 [26] and the USDA My Pyramid Equivalents Database 2.0 [27] to convert the entered information into nutrient and food group intake estimates. For this project, the ASA24-Kids-2012 tool was modified to be interviewer-assisted. RAs operated the platform while asking participants the on-screen questions. An interviewer-assisted approach was used in order to standardize entry of local dishes and common school meals. Trained dietetic interns identified common local dishes from pilot data and school menu reviews to develop standardized recipes for common mixed dishes. These recipes were used during data collection to standardize data entry.

Kilocalories were computed and food groups, as defined by the USDA My Pyramid, were assigned through the ASA24-Kids-2012 platform. The food groups included: fruit (cups), grains (ounces); vegetables, including legumes (cups); dairy (cups); and animal meat (cooked ounces). Additionally, information was collected through a student intake log. Demographic information (grade level, gender, age and race), as well as the source location of each food item, was recorded and later linked by an identification number to the dietary food intake data. Source locations of foods and beverages were categorized as follows: school cafeteria mainline; school cafeteria SB; school cafeteria a la carte line; bake sale or other special venue at school; school vending machine; school store; home, parents or family; fast food restaurant; other restaurant; food store, other outside sources. Not all sources were available at all schools.

### 2.4. Data Analysis

All analyses were conducted using SAS Institute version 9.3 (SAS Institute, Cary, NC, USA) [28]. Demographic and outcome variables were summarized with frequencies for categorical variables and means and standard deviations (SD) and/or medians and interquartile ranges (IQR) for continuous variables. Race/ethnicity was reported as white, black, Hispanic, Asian or Pacific Islander, American Indian or Alaskan Native, and Other. These categories were collapsed into black (Hispanic and non-Hispanic) and “other” for analysis because of the high proportion of black students. Significance testing to identify demographic differences between school types (SB vs. non-SB) was accomplished using the likelihood ratio chi-square test for categorical data and a two-sample *t*-test for continuous data.

The distribution of dietary data for the single dietary recall was positively skewed so these data were summarized and analyzed using medians and interquartile ranges (IQR). Food group consumption was summed at the individual level, and median intake and IQR for each food group were calculated at an aggregate school level (SB or non-SB group). Data were analyzed using mixed model regression methods with school as a clustering variable to accommodate correlations among students in the same school. Box-Cox transformations were used to normalize data.

For analysis of source locations of F/V, school cafeteria a la carte line, bake sale, other special venue at school plus school vending machine were aggregated to “school, not in cafeteria” and fast food restaurant, other restaurant, food store plus other outside sources were aggregated into “food stores and restaurants.” A final derived category “multiple sources” was created for students who obtained F/V during lunch from more than one source (including cafeteria main line; food stores and restaurants, and home, friend or family). Among those students only from the SB schools who reported consuming any F/V at lunch, the number of F/V consumed during lunch and the median and IQR cup amounts of F/V were calculated for lunch time for each source location.

## 3. Results

The sample consisted of 718 students across both types of schools who completed the 24HDR. At the SB schools a total of 1049 students completed the original survey and were eligible. Of these, 664 students were targeted for the 24HDR (60 per school except for two schools where the target was lower because enrollment was low). Of those, 517 provided active parental consents and 458 provided student assents, with a total of 447 24HDR’s being completed (447/664 = 67.3%). At the non-SB schools, a total of 909 students completed the original surveys at these schools; 540 students were targeted (60 per school). Of those, 287 provided active parental consents and 279 provided student assents, with a total of 271 24HDR’s being completed (271/540 = 50.1%). Students were enrolled in the 24HDR when active parental consents were received, and recruiting continued until the school target was reached or until recruitment was exhausted. Descriptive characteristics by school type (SB schools vs. non-SB schools) are shown in Table 1.

Over 64% of the overall sample was female and approximately 87% was African-American. The majority of the sample was in the 7th and 8th grades (62.6%) and the differences in the proportion of students who participated in the 24HDR between SB and non-SB schools was statistically significant (*p* < 0.0001), with SB schools having a higher relative proportion of students participating than the non-SB schools. The students at SB schools were slightly older than those at the non-SB schools, 14.7 mean years compared to 14.0 mean years (*p* < 0.0001). The demographic characteristics of the 24HDR participants were similar to those of the larger sample of students who completed the surveys [22], except that the proportion of females was higher in the 24HDR group (64.1% compared to the survey group 52.1%). Approximately 15% of all students who participated in the 24HDR reported not eating lunch during the period evaluated by the 24HDR.

Students in SB schools who ate lunch during that period had significantly higher median energy consumption during lunch (452 kcal) than students in non-SB schools (395 kcal) (*p* = 0.0136) (Table 2). This lunchtime energy consumption represented 25.7% of the total kcals consumed over the 24-h-period by students in SB schools, and 20.8% of energy consumed during the full day by students in the non-SB schools (*p* = 0.0510). The only other food category in which a significant difference was noted between SB and non-SB schools was cups of total fruit. More fruit was consumed in the non-SB schools (0.25 cups) vs. the SB schools (0.06 cups) (*p* = 0.0061).

Table 3 is based on reports by students who consumed any F/V at school lunch only. If a student reported eating F/V at any other time during the day, that student was not included in Table 3. Table 3 shows the sources of F/V consumed at lunch by these students as well as the median F/V intake for both types of schools with and without SBs. It is inappropriate to test comparisons across the two types of schools because one type does not have the SB and therefore other sources may compensate for the lack of the SB. A total of 536 students reported eating F/V at lunch across both types of schools. At SB schools, 149 students (46%) reported F/V came from the cafeteria main line (n = 145) or the cafeteria a la carte line (n = 4), while students at non-SB schools reported their F/V sources as cafeteria main line (n = 155) or cafeteria a la carte line (n = 5) for a total of 75%. Only a small percentage of SB school students (n = 19 [6%]) reported eating F/V exclusively from the SB, though there was a high proportion of students who reported consuming F/V from multiple sources in SB schools, including F/V from the salad bar (24%) compared to non-SB school students (9%). Interestingly, the median amount of F/V consumed was higher in the non-SB schools (0.76 cups) compared to the SB schools (0.50 cups).

**Figure 1:**
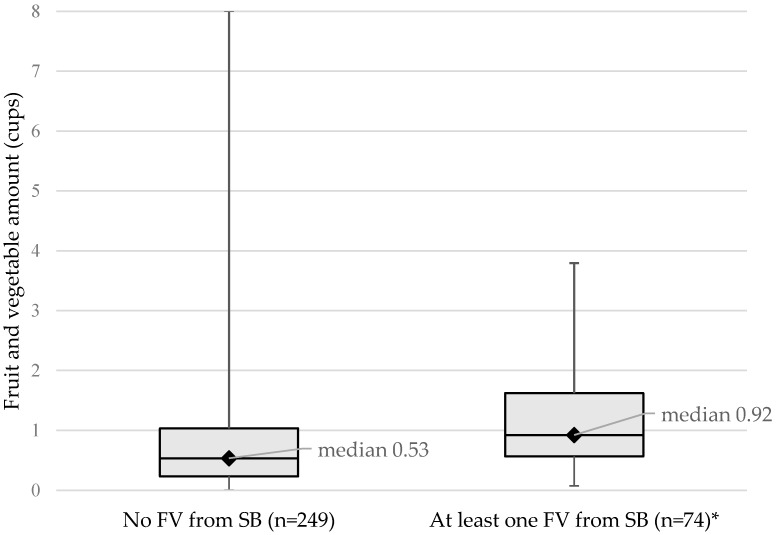
Box-Whiskers Plot showing median fruit and vegetable consumption, range (perpendicular line), Quartile 1 (bottom line of box) and Quartile 3 (top line of box) among students at schools with salad bars (n = 323); * *p* < 0.0001.

When looking at F/V consumption of students at SB schools only (n = 323), the median intake of F/V from students who used the SB was higher (0.92) than that of students whose F/V did not come from the SB (0.53) as depicted in Figure 1. This may be an indication that students who do eat from the SB eat more F/V.

## 4. Discussion

The reported data were the result of a study to examine student consumption of fruit/vegetables in New Orleans public schools with salad bars compared to student consumption of F/V in schools without SBs, as well as to describe F/V consumption among the sample. Overall, almost two-thirds of F/V consumed at lunch across both types of schools was obtained from the school cafeteria. Surprisingly, among students consuming fruit, those from non-SB schools reported a higher amount of fruit consumption at lunch than students at schools with SBs, and there was no difference between the schools in vegetable consumption among those consuming vegetables. It is possible these unexpected findings may have resulted from a lack of environmental factors known to influence student use of SBs [29,30,31,32,33], such as a variety of quality produce, and attractive displays and signage [29,30]; therefore, a more in-depth study is required to determine the influence of these kinds of factors on student choice and consumption. It has previously been reported that increased variety of F/V leads to increased consumption [30], and inclusion of an examination of foods stocked in the SB would provide greater understanding of differences in consumption and would have implications for best practices relative to SB use [31]. While presence of an SB was the key factor in the present analysis, differences in how the SB was implemented by the school was not assessed.

Students have many options within school and from external sources to obtain food items for lunch. In this study, the cafeteria main line was the primary source of F/V items consumed during lunch for both schools with and without SBs. It is possible that students preferred to add F/V to other lunch items, rather than eat F/V exclusively. In this case, the main food line would be more convenient and offer this option to the student. It should be noted, however, that students who obtained at least one F/V from SBs during lunch consumed almost one-half cup more F/V than students at SB schools who did not identify F/V consumed from the SB. Locations outside of the school environment also contributed to F/V consumption, more so for students in the SB schools compared to students in non-SB schools. It is possible that increased awareness and accessibility of F/V via the presence of an at-school lunch SB could generalize to increased F/V consumption from sources external to the school. According to Terry-McElrath et al. [21], SB availability and accessibility were positively associated with middle school green vegetable consumption overall.

Although the SB did not appear to play a major role in F/V consumption at lunch, it should be noted that a sizeable proportion of total 24-h fruit intake (17.5%) and vegetable intake (23.3%) among students was consumed during lunch, suggesting that school lunch is an important contributor to overall daily F/V intake. It should be noted that 15% of the students participating in the 24HDR reported that they did not eat lunch in the reported 24-h period under evaluation. This is concerning inasmuch as youth skipping lunch is not a healthy option, potentially missing essential nutrients on those days, and schools need to be aware of this so that this concern can be addressed.

This study has several limitations. Using a single 24HDR covering just one day for each student instead of multiple days may not reflect accurately the usual intake at the individual level, although it is usually sufficient when analyzing median levels of intake at the group level [34,35,36]. Additionally, the analysis does not take into account the day-to-day variation of food items available to students through the school lunch. Dietary recalls are also limited in assessing actual dietary intake, as they rely on participant memory and over- or under-estimation of amounts of food consumed may occur [37,38]. In addition, while the sample size for this study is acceptable, the percentage of participants is much less than the actual number of students eligible to participate (although the percentage was over 50% for both types of schools), and while randomization did not occur all students who provided an active parental consent were enrolled in the study. Still, participant bias is a possibility.

When considering the cost and time involved in conducting plate waste studies, interview-assisted multiple pass dietary recalls are considered the preferred method among dietary assessment tools [26,35], as was used in this study. A recent validation study on the ASA24-Kids method for collecting dietary recall data for use among adolescents produced poor results compared to interviewer-administered recalls [38]. The present study paired the online platform with an interviewer-assisted method. Although response bias is always a possibility, this method may have contributed to improved data collection over students using the online platform without assistance. This method also allowed us to standardize mixed dishes served at school as well as dishes that are common in the New Orleans area. Additionally, the 24HDR allowed us to identify the relative contribution of lunch to the total 24-h intake, as well as source information for the foods consumed.

As noted in the results, the majority of the dietary recalls were obtained from 7th and 8th grade students, indicating a lower response rate among high school students where total enrollment was higher. An active parental consent was needed for the dietary recall, and the difficulty of obtaining active parental consent with high school students is well known. This may have limited generalizability of the findings. It should be noted that the procedures and incentives for recruitment were standardized across all schools, and the demographic make-up of the sample was similar to the eligible pool of students.

Very few studies have focused on school SB use among middle and high school students. Although the samples were demographically similar across school types, other school-level factors may have influenced student diets and confounded the results. Although it is recognized that there are some cross-sectional designs that would allow for prediction, the cross-sectional design for this study was limited to associations only. There is need for future studies to assess student F/V consumption before and after implementation of SBs in school lunch and to have a longer follow-up period in which the impact of marketing and promotion of the SB and F/V contained on the SB can be captured.

## 5. Conclusions

This study adds to the existing literature examining the effectiveness of school-based SBs as a way to increase consumption of F/V and support a healthier dietary pattern among students in schools where they consume a large proportion of their daily caloric intake. Few studies have examined school SB use and F/V consumption, and most have focused on elementary schools on the West Coast [19,20]. The present study is the first to examine this relationship among mostly African-American middle and high school students living in the urban South.

In the SB schools, an additive effect in consumption would be expected because of F/V offered in the cafeteria main line plus F/V offered on the SB structure. This was not observed and it is possible that F/V consumed from the SB could simply be displacing F/V that would ordinarily have been obtained from the main line. Students who had access to school SBs and consumed at least one F/V from the SBs, had significantly higher median F/V consumption at lunch compared to students who did not consume any F/V from the SB. It cannot be determined from this study whether students who consumed more F/V chose to select F/V because of the presence of the SB, or if they would have selected F/V from another venue without the presence of the SB. SBs have the potential to be effective sources of F/V during school lunch, and studies evaluating the use of an SB along with nutrition education and promotional strategies would add considerably to the literature. F/V marketing and promotion strategies for F/V served through both the main line and the SB are promising environmental additions to the inclusion of the SB in school lunch to increase fruit and vegetable consumption.

## Figures and Tables

**Table 1:** Descriptive characteristics of adolescents in Grades 7–12 in New Orleans, LA, public schools participating in the 24-h dietary recall (24HDR) (n = 718).

Variable	SB Schools	No SB Schools	All Schools
	n (%)	n (%)	n (%)
	447 (67.3) ^1^	271 (50.1)	718 (100)
Gender			
Female	287 (64.2)	174 (64.4)	461 (64.1)
Male	160 (35.8)	96 (35.6)	256 (35.7)
Race/ethnicity			
Black	383 (86.7)	244 (91.0)	627 (88.3)
Other races	59 (13.3)	24 (9.0)	83 (11.7)
Grade level ^1^			
7	123 (27.5)	106 (39.1)	229 (31.9)
8	157 (35.1)	116 (42.8)	273 (38.0)
High School	167 (37.4)	49 (18.1)	216 (30.1)
9	33 (7.4)	19 (7.0)	52 (7.2)
10	34 (7.6)	7 (2.6)	41 (5.7)
11	36 (8.1)	11 (4.1)	47 (6.6)
12	64 (14.3)	12 (4.4)	76 (10.6)
	Mean (standard deviation)		
Age (years)	14.70 ^2^ (1.93)	14.00 (1.55)	14.42 (1.83)

^1^ *p* < 0.0001 (Likeihood ratio chi square); ^2^ *p* < 0.0001 (2 sample *t*-test).

**Table 2:** Median food group and caloric consumption by students during lunch period and proportion from school lunch compared to full day, reported in 24HDR (n = 611).

Food Groups	Students in All Schools n = 611	Students in Schools with Salad Bars n = 379	Students in Schools without Salad Bars n = 232	*p* ^1^
	Median (IQR)	Median (IQR)	Median (IQR)	
Total fruit (n)	524	308	216	
cup	0.17 (0.64)	0.06 (0.48)	0.25 (0.80)	0.0061
% from lunch	17.6 (63.7)	7.0 (62.2)	30.5 (67.7)	0.0357
Total vegetable (n)	589	362	227	
cup	0.25 (0.68)	0.32 (0.73)	0.23 (0.55)	0.6690
% from lunch	25.0 (56.8)	26.8 (65.1)	22.1 (49.5)	0.4686
Total grain (n)	610	378	232	
oz	1.24 (1.50)	1.38 (1.75)	0.98 (1.51)	0.1267
% from lunch	22.6 (29.5)	24.4 (32.6)	18.5 (25.3)	0.1037
Total dairy (n)	582	361	221	
cup	0.45 (0.99)	0.50 (0.97)	0.30 (1.00)	0.0737
% from lunch	31.7 (58.1)	36.1 (59.7)	26.6 (51.7)	0.0628
Total protein (n)	578	357	221	
oz	0.95 (1.92)	0.99 (1.98)	0.81 (1.49)	0.8805
% from lunch	24.2 (53.1)	22.9 (52.4)	26.4 (47.7)	0.9576
Energy consumed (n)	611	379	232	
kcal	432 (340)	452 (319)	395 (358)	0.0136
% from lunch	23.9 (18.8)	25.7 (19.5)	20.8 (17.3)	0.0510
% Students not eating lunch	15.9	15.7	16.2	0.8572

^1^ *p* values are for salad bar schools vs. non-salad bar schools (2 sample *t*-test).

**Table 3:** Sources and median intake of cups of F/V consumed at lunch by students who consumed any F/V at lunch (n = 536).

Food Source	Schools with Salad Bars		Schools without Salad Bars	
	n = 323		n = 213	
	n (%)	Median ^1^ (IQR)	n (%)	Median ^1^ (IQR)
Cafeteria main line	145 (45)	0.50 (0.74)	155 (73)	0.76 (0.90)
Cafeteria a la carte line	4 (1)	1.10 (0.40)	5 (2)	0.64 (0.21)
Cafeteria salad bar	19 (6)	0.56 (0.64)	-	-
School, not in cafeteria	15 (5)	0.23 (0.38)	1 (<1)	0.15 (0)
Home, friends or family	31 (10)	0.42 (0.68)	13 (6)	0.44 (0.96)
Food stores and restaurants	29 (9)	0.60 (0.59)	19 (9)	0.75 (0.91)
Multiple sources	80 (24)	0.99 (1.08)	20 (9)	1.10 (0.90)

^1^ Median cups of fruits and vegetables consumed per person on a given day from that source.
